# Complete mitochondrial genome sequence of *Squalidus chankanensis tsuchigae* (Cypriniformes: Cyprinidae)

**DOI:** 10.1080/23802359.2019.1703564

**Published:** 2020-01-08

**Authors:** So Young Park, Sang Ki Kim, Jin Sagong, Shi-Hyun Ryu, Jeong-Nam Yu

**Affiliations:** Animal and Plant Research Department, Nakdonggang National Institute of Biological Resources, Sangju-si, South Korea

**Keywords:** Mitochondrial genome, phylogenetic analysis, *Squalidus chankanensis tsuchigae*

## Abstract

We determined the complete mitochondrial genome sequence of *Squalidus chankanensis tsuchigae* (Cypriniformes: Cyprinidae). It is 16,603 bp long, with 13 protein-coding genes, 22 transfer RNAs, 2 ribosomal RNA genes, and 1 control region, which is 927 bp long, located between tRNApro and tRNAphe. The overall base composition is as follows: 29.98% A, 16.86% G, 25.44% T, and 27.72% C, with a slight AT bias. These results provide necessary data for phylogenetic studies on *Squalidus* species.

*Squalidus* (Cypriniformes: Cyprinidae) genus consists of 18 species (Froese and Pauly 2017), widely distributed in East Asia. Of those, *Squalidus chankaensis tsuchigae, Squalidus multimaculatus*, *Squalidus japonicus coreanus*, and *Squalidus gracilis majimae* are distributed in South Korea as endemic species (Kim and Park 2007). As ecological and genetic information about *S. chankaensis tsuchigae* is limited (Kim 1997), we determined its mitochondrial genome sequence to analyze phylogenetic relationship within the genus.

*S. chankaensis tsuchigae* was collected from the Nakdonggang River near Gyeongcheonseom Island (36°44.606′N, 128°26.113′E). The voucher specimen (NNIBR-MPL2017GSC0007) has been preserved at the Molecular Phylogenetics Laboratory collection in Nakdonggang National Institute of Biological Resources, Sangju-si, Korea. The mtDNA sequence was extracted from the whole-genome sequencing data (unpublished data) using the Illumina HiSeq4000 platform by GnCBio (Daejeon, Korea). Totally, 42 Gb raw reads were obtained and assembled using Platanus assembler version 1.2.4 (http://platanus.bio.titech.ac.jp/). A total of 231,108 reads were extracted to reconstruct the mitochondrial genome using Deconseq version 0.4.3 (http://deconseq.sourceforge.net/) and annotated using gsMapper version 2.8 (Roche Inc) with fishMito DB as the reference.

The complete mitochondrial genome of *S. chankaensis tsuchigae* (GeneBank accession no. MK840863) is 16,603 bp long, with 13 protein-coding genes, 22 transfer RNAs (tRNAs), 2 ribosomal RNA, and 1 control region, which is 927 bp long, located between the tRNApro and tRNAphe. The start codon of the 12 protein-coding genes is ATG and that of *COXI* is GTG. Four genes (*ND1*, *COX1*, *ND4L*, and *ND5)* end in the TAA codon. Three genes (*ND4*, *ND1*, and *ND2*) contain TAG as the stop codon, whereas seven genes (*ND2*, *COX2*, *COX3*, *ATPase6*, *ND3*, *ND4*, and *Cytb*) share the incomplete stop codon T or TA. The tRNA sequence is 67–75 bp and has a three-leafed clover structure, except tRNA-Ser^UCG^. Two rRNAs are 12S rRNA (958 bp) and 16S rRNA (1688 bp). The overall base composition is as follows: 29.98% A, 16.86% G, 25.44% T, and 27.72% C, with a slight AT bias (55.41%).

To examine the phylogenetic position of *S. chankaensis tsuchigae*, the mitochondrial genome sequence of five *Squalidus* species was downloaded from the GeneBank. The phylogenic tree was constructed using the neighbor-joining (NJ) method with the sequence of the 13 protein-coding genes. A close relationship was observed between *S. gracilis* and *S. chankaensis tsuchigae* (KT948082.1 and in this study) (Figure 1). Reports on ecology, phylogeny, and genetic studies on *S. chankaensis tsuchigae* in English are limited; thus, further studies are necessary to establish more information. Our results will help elucidate the phylogenetic relationship among *Squalidus* species.

**Figure 1.**
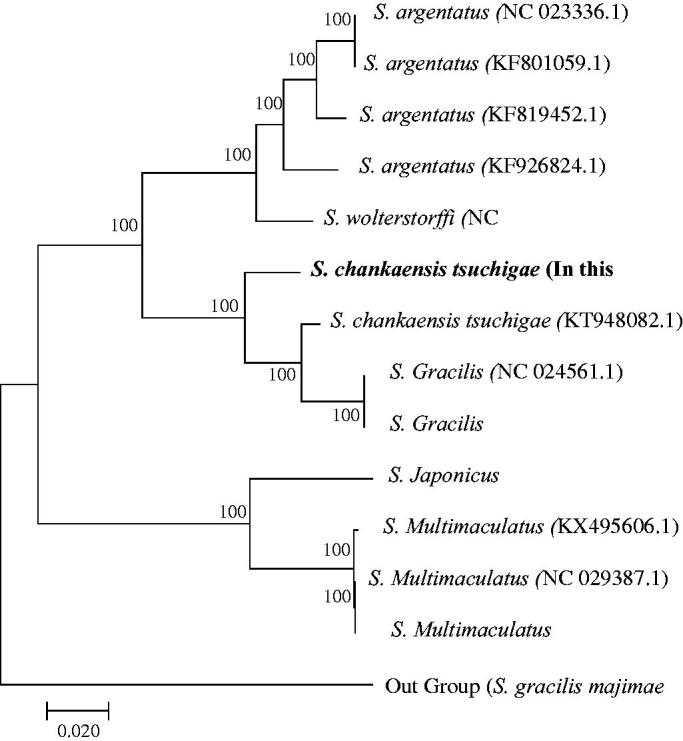
Phylogenetic relationships among five *Squalidus* species based on the 13 coding gene sequence of complete mitochondrial genome sequences in the GenBank were inferred using the neighbor-joining (NJ) method. The numbers at the nodes are bootstrap values computed using 10,000 replications and Kimura’s 2-parameter distance model. The scale bar indicates 0.02 substitutions per nucleotide position.

## References

[CIT0001] Froese R, Pauly D, editors. 2017. FishBase. World Wide Web Electronic Publication. version (02/2017). www.fishbase.org.

[CIT0002] Kim IS. 1997. Illustrated encyclopedia of fauna and flora of Korea. Vol. 37. In: Freshwater fishes. Ministry of Education; p. 518. (in Korean)

[CIT0003] Kim IS, Park JY. 2007. Freshwater fishes of Korea. Seoul, Korea: Kyohak Publishing; p. 499. (in Korean).

